# The Catalyzing Effect of Aggregates on the Fibrillation Pathway of Human Insulin: A Spectroscopic Investigation During the Lag Phase

**DOI:** 10.3390/ijms26157599

**Published:** 2025-08-06

**Authors:** Giorgia Ciufolini, Alessandra Filabozzi, Angela Capocefalo, Francesca Ripanti, Angelo Tavella, Giulia Imparato, Alessandro Nucara, Marilena Carbone

**Affiliations:** 1 Department of Chemical Science and Technologies, University of Rome Tor Vergata, Via della Ricerca Scientifica 1, 00133 Rome, Italy; giorgia.ciufolini@uniroma2.it (G.C.); carbone@uniroma2.it (M.C.); 2 Department of Physics, Tor Vergata University of Rome, Via della Ricerca Scientifica 1, 00133 Rome, Italy; alessandra.filabozzi@roma2.infn.it; 3 Department of Physical and Chemical Sciences, University of L’Aquila, Via Vetoio, 67100 L’Aquila, Italy; angela.capocefalo@univaq.it; 4 Department of Life and Environmental Sciences, Marche Polytechnic University, Via Brecce Bianche, 60131 Ancona, Italy; f.ripanti@staff.univpm.it; 5 Department of Physics, Sapienza University of Rome, P.le A. Moro 5, 00185 Rome, Italy; angelo.tavella@uniroma1.it (A.T.); giulia.imparato96@gmail.com (G.I.)

**Keywords:** amyloid fibrils, insulin, secondary nucleation, lag phase

## Abstract

The kinetics of insulin aggregation and fibril formation were studied in vitro using Scanning Electron Microscopy (SEM) and Fourier Transform Infrared (FTIR) spectroscopy. Our investigation centered on the protein’s morphological and structural changes to better understand the transient molecular configurations that occur during the lag phase. SEM images showed that, already at early incubation stages, a network of disordered pseudo-filaments, ranging in length between 200 and 500 nanometers, develops on the surface of large aggregates. At later stages, fibrils catalyzed by protein aggregates were observed. Principal Component Analysis (PCA) of the FTIR data identified signatures of intramolecular β-sheet secondary structures forming during the lag phase and at the onset of the exponential growth phase. These absorption bands are linked to secondary nucleation mechanisms due to their transient nature. This interpretation is further supported by a chemical equilibrium model, which yielded a reliable secondary nucleation rate constant, K_2_, on the order of 10^4^ M^−2^ s^−1^.

## 1. Introduction

The conversion of soluble proteins into insoluble aggregates remains one of the least understood phenomena in the physics and chemistry of biological macromolecules. Gaining insight into these processes extends beyond their importance in basic science, with potential applications in pharmacology, diagnostics, and preventive medicine. This knowledge can contribute to addressing some of the most prevalent degenerative diseases worldwide, including Alzheimer’s disease (involving Aβ 39–42 peptides and tau protein), Parkinson’s disease (linked to α-synuclein), and systemic amyloidosis [1,2].

The growth of disordered aggregates and/or amyloid fibrils has been extensively studied through both in vitro and in vivo experiments, utilizing spectroscopic techniques like fluorescence, turbidity, nuclear magnetic resonance, and optical and infrared spectroscopy [3,4,5,6]. Each biochemical and biophysical technique focuses on specific aspects of aggregate and fibril formation, so a comprehensive understanding of these kinetics is still lacking. Fluorescence, dynamic light scattering, and turbidity assays primarily focus on the bulk growth of fibrils, leaving molecular-level information to be inferred a posteriori by suitable models. Despite these intrinsic limitations, the use of increasingly high-performance computing tools has enabled access to information on a microscopic scale [7]. Nonetheless, open issues remain: among them, the disclosure of the molecular processes occurring in the early phase of fibrillation is of paramount importance. In the early phase, proteins begin to unfold, losing their secondary and tertiary structures, and form small oligomers that serve as seeds for the development of larger, disordered aggregates and amyloid fibrils. Since these structural transformations and intermolecular interactions are challenging to observe with experimental techniques, this stage is commonly referred to as the lag phase from a phenomenological perspective. It is characterized by a latency period whose duration depends on environmental factors such as pH, concentration, and buffer composition [8], lasting until the onset of the exponential growth phase, during which fibril mass increases rapidly. The lag phase is therefore a “blind” time interval for low-sensitivity techniques but is pivotal for establishing the molecular interactions that trigger the subsequent kinetics [9]. Moreover, the insoluble aggregates formed in the early phase of aggregation pathways, referred to as oligomers, are themselves addressed as cytotoxic molecular species [10].

Fibril proliferation is well known to occur through three mechanisms: primary nucleation, fragmentation, and secondary nucleation [11,12]. Primary nucleation refers to all events in which seeds form and, through the recruitment of monomers, generate mature fibrils through elongation. Fragmentation consists of the breaking of long fibrils into smaller ones, which in turn can elongate, and is activated when the concentration of fibrils becomes significant. Secondary nucleation is a surface-catalyzed process in which, during interaction with monomers, the surface of pre-existing fibrils or proto-fibrils templates additional seeds. It is commonly accepted that the formation of seeds by secondary nucleation is responsible for the sudden increase observed in the growth phase. Observing these mechanisms in the lag phase and at the onset of the growth phase is difficult even through Raman, NMR, and infrared spectroscopy, and most attempts have been unsuccessful or have yielded unclear results.

Since the discovery of insulin in 1921, efforts to detail the structural and morphological changes of this macromolecule along the path to aggregation and fibrillation have been only partially successful. Indeed, a thorough understanding of in vivo and in vitro kinetics of aggregates is an essential requirement for pharmaceutical applications and production and is crucial for the prognosis and treatment of diabetic patients [13]. As is known, insulin is a 51-amino-acid protein formed by two chains, A and B, bonded through two disulfide bonds (A7-B7 and A20-B19), which confer a globular shape to the molecule. Produced by the pancreas, this hormone regulates glucose metabolism in healthy subjects. Insulin accumulation is responsible for localized amyloid deposit in diabetic patients [14]. Among amyloid proteins, insulin is perhaps the least understood in terms of the microscopic mechanisms driving its fibrillation. Experimentally, there is a large variety of behaviors which describe the insulin fibril dynamics, ranging from sigmoidal time courses, typically assigned to secondary nucleation processes, to hyperbolic or exponential trends where primary nucleation is mainly involved [15]. Understanding the pathways differences at the molecular level—beyond staining protocols—is highly desirable, as the aggregation of this hormone poses significant challenges in both pharmacology and medicine.

In this study, we investigate the fibrillation of human insulin monomers using scanning electron microscopy (SEM) and Fourier transform infrared spectroscopy (FTIR) to analyze the catalytic properties of protein aggregate surfaces, which play a key role in promoting and initiating secondary fibril nucleation. SEM imaging assisted by Dynamic Light Scattering experiments is essential for morphologically distinguishing fibril formation (on-pathway regime) from aggregate formation (off-pathway) and can provide semi-quantitative data to complement spectroscopic analysis. FTIR experiments, combined with multivariate analysis, will highlight secondary structure changes occurring during the lag phase and near the growth phase. According to the literature, human insulin at concentration of 1 mM/L in aqueous solution and at pH 2, presents a lag phase lasting around 2 h when incubated at 65 °C [16]. During this period, fragmentation and secondary nucleation processes are expected to compete with other mechanisms such as primary nucleation and polymerization. Our approach goes beyond the level of information provided by Tioflavin T (ThT) assay experiments, as the latter do not enter the molecular mechanisms preceding the exponential growth phase. By studying in detail the secondary and tertiary structures of the molecules involved in the interaction processes during incubation in the lag phase, we aim to disclose the nature of the nucleation processes and quantify their relevance in the aggregation pathway. The results of this research emphasize the key role of surface–monomer interactions in insulin fibrillation, aligning with the secondary nucleation model.

## 2. Results and Discussion

### 2.1. Morphological Assessment

**Figure 1:**
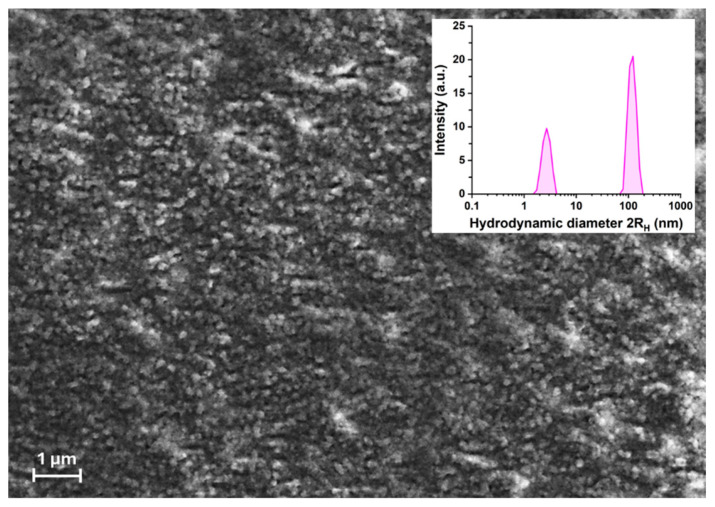
SEM image at time zero: small oligomers representing the early oligomeric state. The inset shows the hydrodynamic diameter (2R_H_) from DLS measurements of the insulin solution prior to incubation. Two populations (purple histograms) are observed, with 2R_H_ values of 2.7 ± 0.5 nm and 119.0 ± 20.0 nm, each with a polydispersity index (PDI) of 0.03. Size distribution was determined by intensity-weighted analysis using the NNLS algorithm.

To establish a reference point for the aggregation process, the insulin sample was first analyzed at time zero, prior to incubation. SEM images of this initial state (Figure 1) reveal the presence of small, approximately spherical aggregates with an average diameter around 100 nm. However, the presence of smaller aggregates, potentially undetectable under these imaging conditions, cannot be excluded. DLS analysis prior to incubation corroborates the findings of the SEM images at time zero, as only two distinct particle populations, with a hydrodynamic diameter 2R_H_ of 3 nm and 120 nm, are observed. The former species likely represents monomers and/or dimers, while the second represents small oligomers.

**Figure 2:**
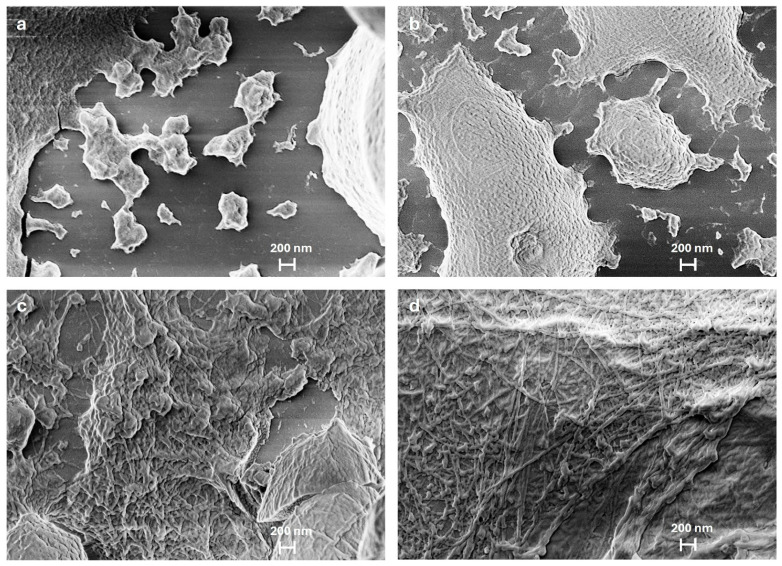
SEM images of insulin incubation at different time points: (**a**) 20 min: Small aggregates with a smooth surface, beginning of protein aggregation; (**b**) 60 min: Increase in aggregate size and in the surface corrugation; (**c**) 100 min: Onset of mature fibril formation; (**d**) 140 min: Elongation of mature fibrils.

SEM data in Figure 2a–d capture the evolution of insulin aggregation into oligomers and fibril formation at 20, 60, 100, and 140 min of incubation. A sample after 20 min of incubation (Figure 2a) reveals isolated aggregates that appear disorganized and scattered, exhibiting irregular shapes with rough surfaces. The roughness arises from a network of disordered pseudo-filaments, ranging in diameter from 200 to 500 nanometers, representing early protofibrillar aggregates. As in the t = 0 case, SEM data were supported by DLS measurements which provide evidence of three populations displaying average diameters of 3, 220 and 820 nm, respectively.

**Figure 3:**
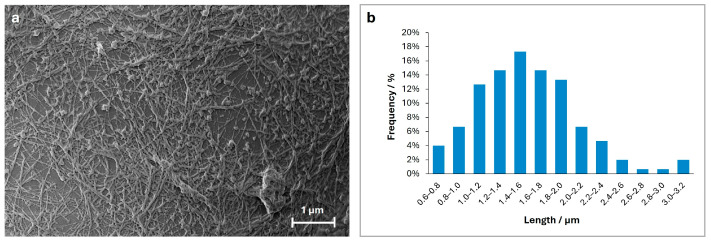
Panel (**a**) Mature fibrils formed after 140 min of insulin incubation. Panel (**b**) Histogram of fibril length distribution, showing the percentage of fibrils within each length interval (measured in μm).

After 60 min of incubation (Figure 2b), the smallest aggregates become less prevalent, while those as large as 1 to 3 µm increase in proportion. Notably, there is significant expansion in the large protofibrillar regions, which now dominate approximately 50% of the observable surface area. Within these regions, the surface corrugations become more evident, suggesting the emergence of fibrillar structures. This stage likely represents a phase in which protofibrils evolve into more organized intermediates, exhibiting greater structural cohesion. At this stage, fibrils remain indistinct, preventing an accurate measurement of their average length and suggesting that fibrillation is still in its intermediate phase. At 100 min of incubation (Figure 2c), individual aggregates are no longer visible, now forming a dense network of protein structures organized within extended regions. Between these assemblies, areas of aggregation displaying a tendency toward elongation can be observed, marking the onset of fibril formation. Finally, at 140 min, SEM images show a dense network of fully formed fibrils, covering a large part of the aggregate surface (Figure 2d). These mature fibrils appear as continuous, elongated structures, often organized into bundles forming intricate patterns. A comprehensive statistical analysis of fibril length distribution was performed at this stage, where the presence of mature fibrils ensures a greater accuracy in statistical characterization. Figure 3a displays the selected region for statistical analysis, with dimensional measurements performed on a representative set of clearly identifiable fibrils. Smaller, disorganized aggregates remain visible and do not progress into mature fibrils. Figure 3b presents a histogram depicting the frequency distribution of fibril lengths ranging from 0.6 to 3.2 μm with an average length of 1.6 ± 0.5 μm. In addition, the fibrils exhibit diameters ranging from approximately 5 to 20 nm, indicating considerable variability. In other studies, insulin fibrils are reported with diameters ranging from 3 to 15 nm and lengths extending to several microns, consistent with our findings. Occasionally, individual fibrils group into bundles of two to five strands, twisting together to form filaments with diameters ranging from 10 to 20 nm, highlighting their intricate hierarchical organization [17,18]. Statistical analysis of the smallest globular oligomers was also conducted, focusing on their diameters. Their sizes range from 50 to 170 nm, with an average diameter of 100 ± 20 nm. A histogram illustrating the frequency distribution of oligomer diameters, similar to that for the fibrils, is provided in the Supplementary Materials (Figure S1.1).

The formation of large aggregates during incubation has been previously documented in the literature [19]. Although their timescale and conditions differ significantly, the number and size of the larger particles continue to grow with incubation time. The SEM images depict clear progression: from initial aggregation, marked by growing protein clusters, to the development of a complex network of interwoven filaments that eventually mature into fibrils. Notably, filaments are found on pre-existing protein layers and on the surfaces of large aggregates, suggesting a surface-catalyzed fibrillation mechanism (Figure S1.2 of the Supplementary Materials). Previous research by Nayak et al. [12] has demonstrated that surface roughness accelerates aggregation and fibrillation, likely by increasing the local concentration of protein molecules. Furthermore, it is known that hydrophobic interfaces play an important role in the monomers’ unfolding and subsequent aggregate formation [20]. Our findings support this assessment, highlighting the role of heterogeneous surfaces in fibril formation dynamics which become observable after 100 min of incubation. Importantly, the exposed β-sheet regions on the aggregate surfaces play a pivotal role in templating new proto-filaments and protofibrils, while also facilitating their intertwining [21].

### 2.2. Phenomenology of the Aggregation Pathways by FTIR

Having observed the morphological changes in insulin aggregates at different stages of fibrillation through electron microscopy, we now move to the investigation of the molecular mechanisms underlying the fibrillation kinetics by FTIR. In the formation of insulin fibrils, two types of kinetic pathways are usually considered. The first scenario describes the progression of fibril content over time following a sigmoidal curve, with clearly defined lag, growth, and stationary phases. In contrast, the second scenario follows a hyperbolic curve of fibril percentage, without a clear separation of the different structural phases. The dominance of one kinetic pattern over the other is determined by multiple factors, including environmental conditions and the intrinsic structural characteristics of the protein molecule. Performing experiments, we observed both sigmoidal and hyperbolic kinetic profiles occurring with similar frequency; however, the hyperbolic pattern offers considerably less information compared to the sigmoidal one. Therefore, the following discussion centers on the FTIR data displaying sigmoidal kinetics, while a comparable analysis of the hyperbolic kinetics is presented in the Supplementary Materials (S2).

**Figure 4:**
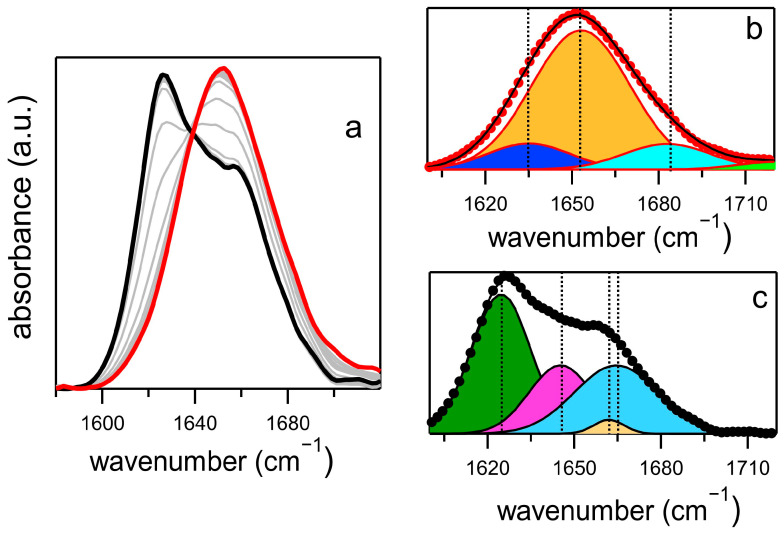
(**a**) Time evolution of the amide I band of human insulin. The absorption spectrum at t = 0 is reported in red, that acquired after 4 h in black. Gray curves represent absorption spectra at intermediate times. (**b**) Gaussian deconvolution of the t = 0 spectrum. The orange-filled curve represents the α-helix contribution, the cyan one the loops and turns secondary structures and the blue curve the β-sheet contribution. (**c**) Spectral deconvolution at the final stage of incubation. Green and purple filled curves represent the fibril β-parallel and the random coil secondary structures, respectively. Vertical dashed lines in panels (**b**,**c**) indicate the central frequencies of the Gaussian contributions.

An example of an FTIR absorption spectrum in the amide I region (C=O backbone stretching vibration) is illustrated in Figure 4a. Gaussian deconvolutions of spectra at the initial (t = 0) and final (t = 4 h) steps are shown in panels b and c, respectively. At t = 0, the Gaussian fit provides secondary structure percentages that align with those reported in databases and previous literature [16,22]. The insulin monomer comprises three α-helical segments, one β-turn structure spanning from B20Gly to B23Gly, and a disordered B-chain C-terminus ranging from B24Phe to B30Thr. The normalized area of the Gaussian components indicates a 76% α-helix secondary structure (Gaussian centered between 1650–1655 cm^−1^), 12% turns and loops (1675–1685 cm^−1^), and 12% spectral intensity within the 1630–1638 cm^−1^ region, attributable to the intermolecular β-sheet of the B chains paired to form dimers [23,24].

In FTIR spectroscopy, β-sheet contributions typically display a broad and variable frequency distribution. This variability is governed by the complex interplay of two primary mechanisms. First, Transition Dipole Moment delocalization tends to red-shift the absorption band while also narrowing its width, reflecting increased structural order and extended coupling across β-strands. Conversely, environmental disorder—arising from heterogeneous local environments, conformational variability, or solvent interactions—induces a blueshift and broadens the band, indicative of more localized vibrational modes and diminished inter-strand coherence [25,26]. The balance between delocalized and local modes can be inferred by the central frequency and amplitude of these contributions, thus aiding in assigning the origin of these absorptions.

The deconvolution of the spectrum at the final incubation stage, shown in panel c, presents a markedly different pattern. The most intense contribution at 1625 cm^−1^ (44% of the total intensity) originates from β-parallel strands bundled into fibrils. The contribution at 1664 cm^−1^ (31%) is attributable to unordered secondary structures from oligomers [23]. However, this assignment remains debated in the literature, as β-turn moieties may also contribute spectral intensity in the same region [24]. Clear evidence of a random coil secondary structure is provided by the band at 1645 cm^−1^ (23%), serving as a marker for unfolded molecules not involved in the aggregates. Finally, a small contribution (2%) to the amide I band appears around 1660 cm^−1^, attributed to remnants of the α-helical secondary structure in unbound molecules.

### 2.3. Principal Components Analysis (PCA) of the FTIR Data Set

Since Gaussian spectral deconvolution can produce ambiguous outcomes when applied to broad, rapidly evolving spectra, we instead employed Principal Component Analysis (PCA) on the second derivative of each absorption spectrum. The use of the second derivative enhances the detection of subtle spectral variations that may be obscured in the original data, while PCA—an unsupervised statistical method—offers a robust protocol overcoming the limitations associated with traditional deconvolution techniques. Referring to established protocols [27,28], the second derivative spectrum at time t, $S\left(\omega ,t\right),$ can be expressed as a linear combination:

(1) $$S\left(\omega ,t\right)\;=\;\sum_{i}s_{i}\left(t\right)L_{i}\left(\omega \right)$$

where $s_{i}\left(t\right)$ are the weights (scores) of the $L_{i}\left(\omega \right)$ eigenspectra (loadings) for the dataset of time-dependent original spectra. The time evolution of the second derivative is then determined by the sequence of the scores, while the loading functions highlight the most significant spectral variations.

**Figure 5:**
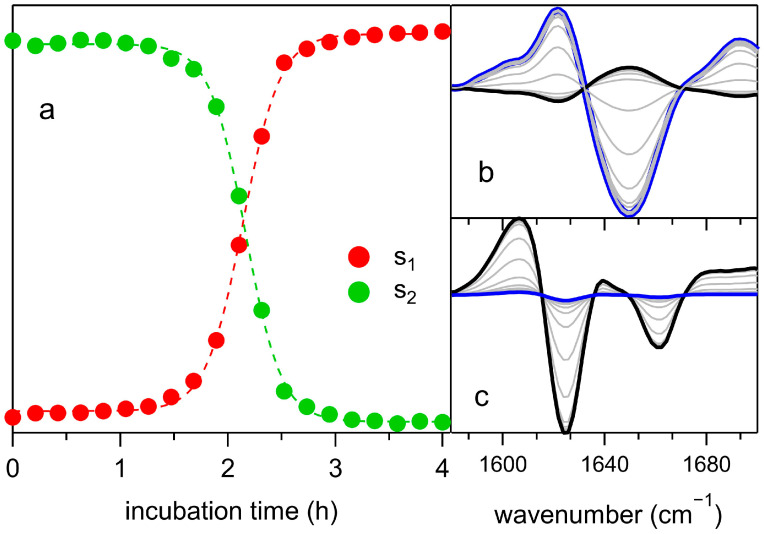
PCA analysis of the FTIR spectra. (**a**) Scores s_1_ (red) and s_2_ (green) reported vs. time. Fit to data are performed according to Equation (3). (**b**,**c**) Time evolution of the quantities $s_{2}\left(t\right)L_{2}\left(\omega \right)$ (**b**) and $s_{1}\left(t\right)L_{1}\left(\omega \right)$ (**c**), respectively. The blue spectrum corresponds to the initial state, the black one to the final. Gray spectra refer to intermediate incubation times.

When applying PCA expansion to the spectra reported in Figure 4, the overall time behavior is primarily explained by the lowest orders *i* = 1, 2 which together account for 99% of the variance in the entire set of second derivatives. Thus, Equation (1) can be rewritten as:

(2) $$S\left(\omega ,t\right)=s_{1}\left(t\right)L_{1}\left(\omega \right)+s_{2}\left(t\right)L_{2}\left(\omega \right)+R\left(\omega ,t\right)$$

where $R\left(\omega ,t\right)$ is the residual part of the expansion and represents the total contribution of the highest order terms. Scores $s_{1}$ and $s_{2}$ as functions of incubation time (reported in Figure 5a) closely reproduce the sigmoidal trend typically observed in fluorescence experiments for fibrillar proteins [29,30] providing insights into the percentage variation in fibrils and monomers. Therefore, the first terms in the expansion of Equation (2) reflect the variations in fibrillar and monomer masses, with the corresponding coefficients $s_{1}$ and $s_{2}$ effectively capturing these changes.

Figure 5a indicates that the duration of the lag phase can be estimated as t_lag_ ≈ 1.5 h. The overall trend of the data is well described by a Boltzmann function, with the time of the inflection point of the rise (T*) and the apparent growth rate K = 1/*T*_0_ serving as fitting parameters.

(3) $$s_{1,2}\left(t\right)=\frac{A}{1+\exp\left[-\left(\frac{t-T^{*}}{T_{0}}\right)\right]}+B$$

Fit to data with Equation (3) provided a half-time *T*^∗^ = 2.1 h and an apparent growth rate K = 1/*T*_0_ = 5.9 h^−1^.

The loading *L*_1_ in Figure 5b describes the kinetics of β-secondary structure features (sharp dip at 1625 cm^−1^) and the formation of oligomers (minimum around 1660 cm^−1^). In turn, *L*_2_ (Figure 5c) accounts for the disappearance of the native α-helical secondary structure (1650 cm^−1^) during aggregation. Information on molecular interactions before and during the growth phase can be obtained from the highest-order terms provided by the residual $R\left(\omega ,t\right)$ function. Hereafter, we consider only the third and fourth orders of the expansion, i.e., $R\left(\omega ,t\right)=s_{3}\left(t\right)L_{3}\left(\omega \right)+s_{4}\left(t\right)L_{4}\left(\omega \right)$.

**Figure 6:**
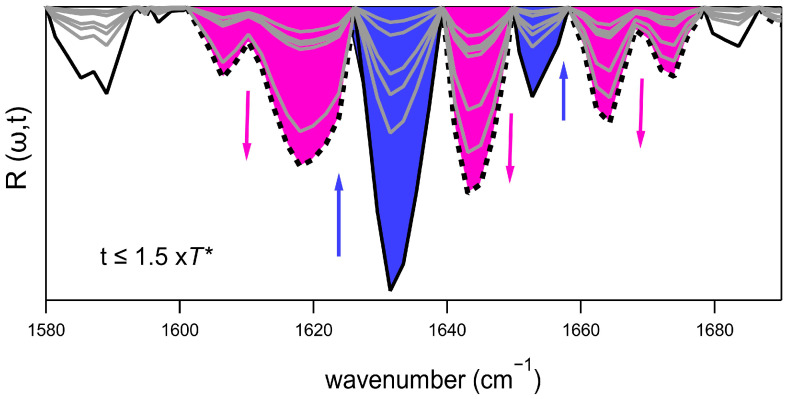
Evolution of the higher orders of the PCA expansion during the lag and the exponential growth phase for incubation times less than 1.5 × T* (T* = fibrillation half-time). The solid black spectrum refers to $R\left(\omega ,t=0\right)\;$ while the dashed black one is $R\left(\omega ,t=1.5\times T^{*}\right).$ The gray spectra refer to $R\left(\omega ,t\right)$ at intermediate times. The arrows indicate the behavior of the minima vs. time. The blue-filled minima highlight the progressive decrease of the spectral contributions, the purple-filled ones the contributions that increase with time.

Figure 6 presents the $R\left(\omega ,t\right)$ function for the same dataset previously discussed. This function captures variations in the second derivative minima, which are attributable to changes in secondary structures over time. In particular, the two minima at 1633 cm^−1^ and 1675 cm^−1^ correspond to parallel and antiparallel β-sheets due to the presence of dimers, and their intensity decreases over time. Similarly, the small minimum at 1650 cm^−1^, assignable to α-helix contributions, also vanishes relatively quickly during the lag phase. Halfway through the fibrillation process, neither dimers nor α-helix contributions are observed in $R\left(\omega ,t\right)$ anymore. Instead, the concomitant formation of β-intramolecular structures (minima between 1605 and 1622 cm^−1^) and random coil contributions (1645 cm^−1^) is observed. These contributions rapidly drop to zero as the system approaches the stationary phase. Additionally, during the entire lag phase, an increase in intensity at 1660 cm^−1^ is observed, attributed to the presence of oligomers and/or turn structures. In the final stationary phase of the incubation, $R\left(\omega ,t\right)$ varies only slightly over time, except for a faint decrease in the minimum at 1633 cm^−1^, indicating the formation of dimers at their final equilibrium concentration.

**Figure 7:**
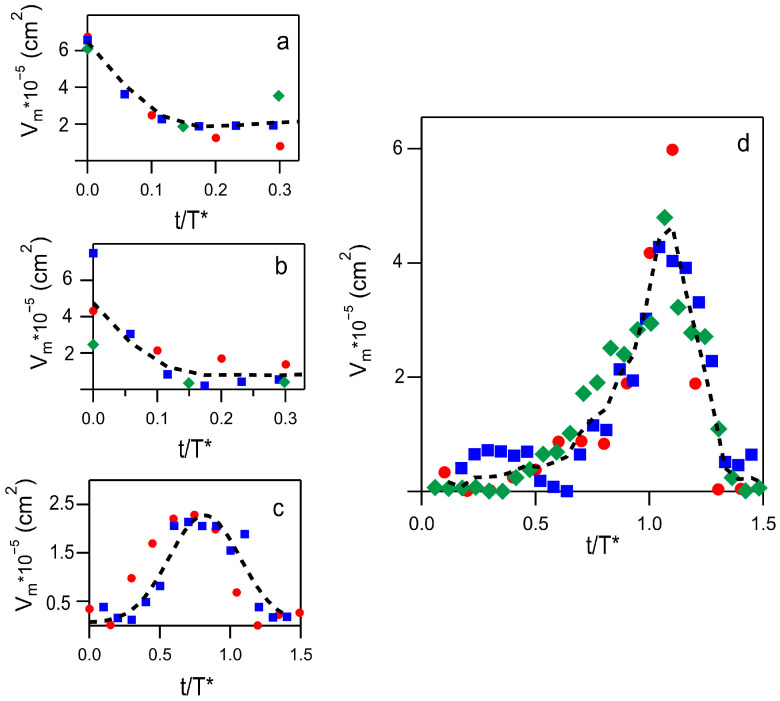
Time evolution of the minima V_m_ (absolute values) as obtained from different experiments and reported with different symbols and colors (green diamonds, red circles and blue squares). (**a**) V_m_ for a-helix secondary structures. (**b**) V_m_ for the dimer’s contribution at 1633 cm^−1^. Fit to data in a and b (dashed curves) provided a damping rate of 0.1T* for both contributions. (**c**) V_m_ data for the oligomer’s contribution. (**d**) V_m_ values obtained in the 1600–1620 cm^−1^ spectral region. The dashed curves in panels c and d are averages of all experimental data.

To make the aggregate assembly/disassembly dynamics more quantitative, it is useful to consider the minimum value, V_m_, of the above-discussed contributions. These values are reported in Figure 7 for three different samples identically analyzed by a PCA approach. To account for slight differences in the T* values, data are analyzed as a function of the variable t/T*. Despite the uncertainties arising from minimal absorption, the time evolution of selected secondary structures remains clearly observable. In panels a and b of Figure 7, V_m_ is reported for the α-helix (1650 cm^−1^) and the dimer contribution (1633 cm^−1^), respectively. Both contributions are damped in the initial phase of incubation, well before the lag phase ends. The reduction in α-helix content is less evident than that shown in Figure 4c and likely reflects an initial step in seed formation. The V_m_ values displayed in Figure 7c, corresponding to the minimum near 1664 cm^−1^, provide insight into the kinetics of oligomer development during the early stages. These values rise throughout the lag phase but drop sharply at the onset of the exponential growth phase. This behavior supports the hypothesis that seeds gradually lose their secondary structure in favor of the extended β-sheet conformation characteristic of mature fibrils, indicating that this parameter may mark the transition from seed to fibril. The most relevant outcome is reported in Figure 7d, which shows the V_m_ values for the β-aggregate band between 1600 and 1620 cm^−1^. The spectral contributions in this region are associated with the formation of intramolecular β-sheet secondary structures originating from oligomers or complexes [31,32]. These low-frequency components exhibit two notable features: (i) they emerge prior to the formation of mature fibrils, and (ii) they reflect transient vibrational states linked to evolving molecular configurations. For these contributions, the V_m_ value reaches its peak around the half-life T* and their temporal behavior mirrors the kinetics of secondary nucleus formation, as previously inferred from detailed analyses of fluorescence kinetic profiles [8,33]. Since the rate of secondary nucleation depends on the presence of both fibrils and monomers, the concentration of secondary nuclei is expected to peak when both species coexist in comparable amounts in the solution. Consistent with the hypothesis that these bands originate from surface-catalyzed processes, we assume their intensity is proportional to the number of monomers binding to existing fibrils. Therefore, the absorption observed in the 1600–1620 cm^−1^ range can serve as an indicator of secondary nucleus formation, under the assumption that all oligomers formed on fibril surfaces function as secondary nuclei upon release. In this framework, the integrated intensity of these absorption bands acts as a marker for the quantity of secondary nuclei at various incubation times.

From the intensity values in Figure 7d, a reliable estimate suggests that 10–30% of insulin molecules are involved in β-intramolecular processes (see Supplementary Materials S3). Since Gaussian deconvolution indicates that at least 44% of the molecules organize into mature fibrils, comparing this with the number of insulin molecules involved in low-frequency absorption—assumed to serve as a marker of secondary nucleation—attests to the high efficiency of this mechanism.

It would be beneficial to correlate the intensity of the observed bands with both the number and size of secondary nuclei formed during the exponential growth phase. Since the early 2000s, models describing the formation and kinetics of primary and secondary nuclei have been developed, motivated by the increasing interest in studying insoluble aggregates of biological molecules [34]. Nonetheless, computational and analytical treatments remain challenging due to the system’s heterogeneity, where the concentrations of nuclei [*n*(t)], fibrils [*M*(*t*)], and monomers [*m*(*t*)] all change dynamically over time. In our experiments, however, the acquisition times are considerably longer than the microscopic relaxation times, allowing us to assume that the equilibrium equation holds for the species in solution:

(4) $$\left[n\left(t\right)\right]={\tilde{k}}_{n}\left[M\left(t\right)\right]{\left[m\left(t\right)\right]}^{n}$$

**Figure 8:**
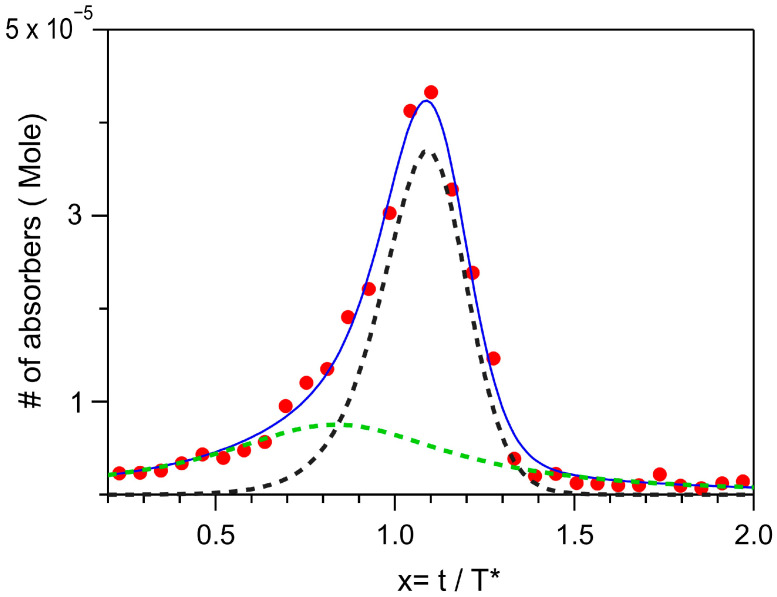
Average V_m_ curve (red dots) together with the best fit to data (blue line). The green dashed curve is a Lorentzian line; the black dashed line is a curve according to Equation (4) with *n* = 2. For the details of the fitting procedure, refer to Supplementary Materials.

In Equation (4), the constant ${\tilde{k}}_{n}$ represents the ratio between the rate of nuclei formation and their dissociation. Notably, the order *n* in Equation (4) does not necessarily correspond to the number of monomers forming nuclei; rather, it can be determined from macroscopic observations, such as T* and concentration [7]. However, the value *n* = 2 has been considered the most relevant order for promoting nucleation in the case of Aβ42 [27], as it accurately reproduces experimental data and provides reliable rate values. Following this approach, we used Boltzmann curves for [*M*(*t*)] and [*m*(*t*)] to fit the experimental data in Figure 7d (Supplementary Materials, S4) while setting *n* = 2. In Figure 8, an example of the best fit to the data is shown. The asymmetry of the experimental curve is likely due to absorption processes unrelated to surface-promoted effects, such as contributions from oligomers at t < T* and accounted in the fit with a Lorentzian contribution. The fitting procedure yields an average value of T*/T_0_ = 11, which is in good agreement with the phenomenological value obtained from Figure 5 and aligns with data from the literature [27]. Additionally, the rate constant for secondary nuclei formation is obtained, K_2_ = 7.3 × 10^4^ M^−2^ sec^−1^. The uncertainty in this value is about 30%, mainly arising from the estimation of the final fibril concentration based on SEM analysis. Despite this uncertainty, the determined rate constant is consistent with that reported for the Aβ42 peptide, indicating that the aggregation mechanisms are likely similar in both systems.

## 3. Materials and Methods

### 3.1. Sample Preparation

Lyophilized human insulin was purchased from Merck (Darmstadt, Germany) (REF. 11376497001). The solutions were prepared by dissolving 5.8 mg of powder in 1 mL of H_2_O for SEM experiments or D_2_O for FTIR. pH and pD were adjusted to 2. Vials were incubated at 65 °C using either a thermo-block or by immersion in a thermal bath. The zero-incubation time point (T_0_) corresponds to the freshly prepared sample, prior to any thermal treatment. At this stage, all measurements were carried out at room temperature (25 °C), before starting incubation at 65 °C.

### 3.2. SEM Imaging

The morphology of fibrillated insulin samples at different incubation times (20, 60, 100, and 140 min) was analyzed using scanning electron microscopy. Silicon wafers were used as substrates for sample preparation. The wafers were cleaned by sonication in ethanol and dried under a nitrogen stream. A 10 µL drop of insulin solution was deposited onto a silicon wafer at each incubation time, and to minimize drying artifacts, the excess liquid was gently removed with filter paper. Imaging was performed using a SUPRA™ 35 Field Emission Scanning Electron Microscope (Carl Zeiss SMT, Oberkochen, Germany), operating at acceleration voltages of 1.5 kV, using imaging conditions best suited for organic samples, without metallization. SEM images were captured to assess fibril morphology and distribution. Quantitative analysis of fibril lengths was performed using ImageJ v1.54g software, with manual validation [35]. Given the extensive fiber entanglement, the total fibril length was estimated based on the 150 most clearly visible structures.

### 3.3. Dynamic Light Scattering

DLS measurements were performed using a Malvern NanoZetaSizer instrument (Malvern Instruments Ltd., Malvern, UK), equipped with a 5 mW HeNe laser, a Peltier temperature control system, and backscattering detection. The apparent hydrodynamic radius (R_H_) distributions were obtained by analyzing the autocorrelation function of the scattered light intensity. Insulin solutions were directly placed in 1 cm path length quartz cuvettes and analyzed after 0 and 20 min of incubation, with the sample temperature maintained at 65 °C. The hydrodynamic radii size distribution of the various populations potentially present in the solutions and the polydispersity index are determined using standard proprietary algorithms.

### 3.4. FTIR Data Acquisition and Analysis

A Bruker IFS66VS Michelson interferometer (Bruker, Billerica, MA, USA) was used for the FTIR analysis. The instrument was equipped with a KBr beamsplitter and a DTGS detector, with the spectral resolution set at 4 cm^−1^. A custom-built spectroscopic cell (sealed with CaF_2_ windows and featuring a 25 μm optical path) was housed in the interferometer and filled with a 20 μL drop of the solution. The cell temperature could be varied via external liquid circulation. The entire setup was thermalized at 65 °C in less than 5 min. Infrared spectra were acquired overnight at fixed time intervals of 10 and 4 min, while keeping the entire setup under vacuum. Infrared absorption spectra were pre-processed using the Opus software v6.0 for baseline subtraction and normalization [36]. Second-derivative PCA analysis was performed using the NIPALS algorithm from the Parvus package version 0.5.8 [37]. Final data analysis was conducted using Igor Pro v9 software [38].

## 4. Conclusions

The kinetics of insulin aggregation have gained significant attention due to their pronounced variability, with fibril formation exhibiting sigmoidal, convex, or exponential time-dependent behaviors. In the sigmoidal regime, master equation models tend to prioritize fragmentation as the dominant mechanism of fibril proliferation. Nonetheless, secondary nuclei formation and surface-catalyzed effects must also be considered, as previously investigated by Jansen et al. [39] and Manno et al. [40]. In this study, we conducted a morphological analysis of fibrils during incubation using SEM imaging, revealing that their formation is promoted by interactions between molecules and the surfaces of larger aggregates. Similar processes have been shown to enhance fibril proliferation in Aβ42, suggesting their potential significance in insulin aggregation as well. FTIR spectroscopy highlighted absorption bands indicative of transient secondary structural changes during the lag phase. Notably, those persisting until the midpoint of the exponential growth phase were attributed to intramolecular bonds formed during secondary nucleation events. This observation is consistent with SEM results, underscoring the key role of monomer–surface interactions in aggregation kinetics. Further support that FTIR absorptions originate from secondary nucleation is provided by a semi-quantitative analysis of the observed features, from which an affordable value for the secondary nuclei creation rate was deducted. Importantly, FTIR provided novel insights not into fibril mass but into microscopic molecular changes occurring during incubation. While these findings do not fully elucidate the microscopic dynamics of aggregation, they pave the way for a new perspective in which advanced tools can be used to directly verify molecular-level changes during fibril formation.

## Data Availability

Data are available upon request.

## Supplementary Materials

The following supporting information can be downloaded at: https://www.mdpi.com/article/10.3390/ijms26157599/s1.

## Abbreviations

The following abbreviations are used in this manuscript:

SEM	Scanning Electron Microscopy
FTIR	Fourier Transform Infrared Spectroscopy
PCA	Principal Component Analysis
DLS	Dynamic Light Scattering
PDI	Polydispersity Index
