# Supplementary material for: The Catalyzing Effect of Aggregates on the Fibrillation Pathway of Human Insulin: A Spectroscopic Investigation During the Lag Phase
Source: Int J Mol Sci. 2025 Aug 6;26(15):7599. doi: 10.3390/ijms26157599 (PMC12347091; doi:10.3390/ijms26157599)
Supplement: Supplementary file 1 [file ijms-26-07599-s001.zip › ijms-3761081-supplementary.pdf]

Supplementary Material to:

The Catalyzing Effect of Aggregates on the Fibrillation Pathway of Human Insulin: A Spectroscopic Investigation During the Lag Phase

By:

Giorgia Ciufolini, Alessandra Filabozzi, Angela Capocefalo, Francesca Ripanti, Angelo Tavella, Giulia Imparato, Alessandro Nucara and Marilena Carbone

S1. SEM analysis

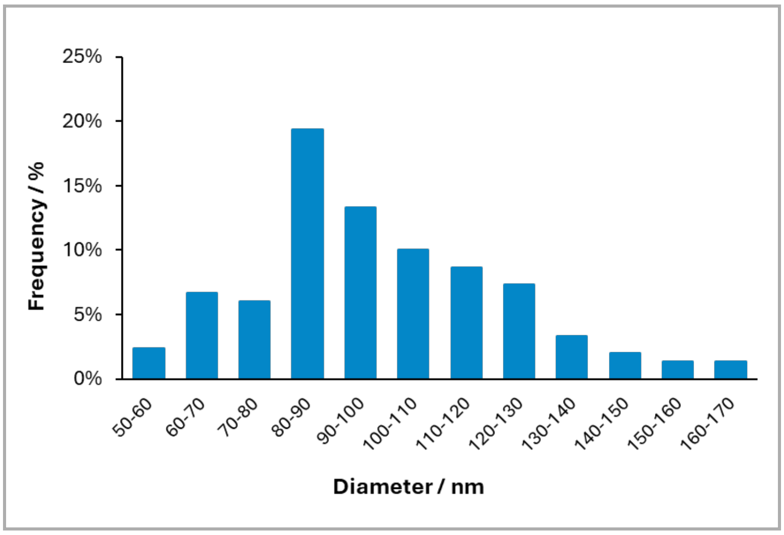

Figure S1.1 Diameter size distribution of the oligomers (measured in nm). Average diameter of 100 ± 20 nm.

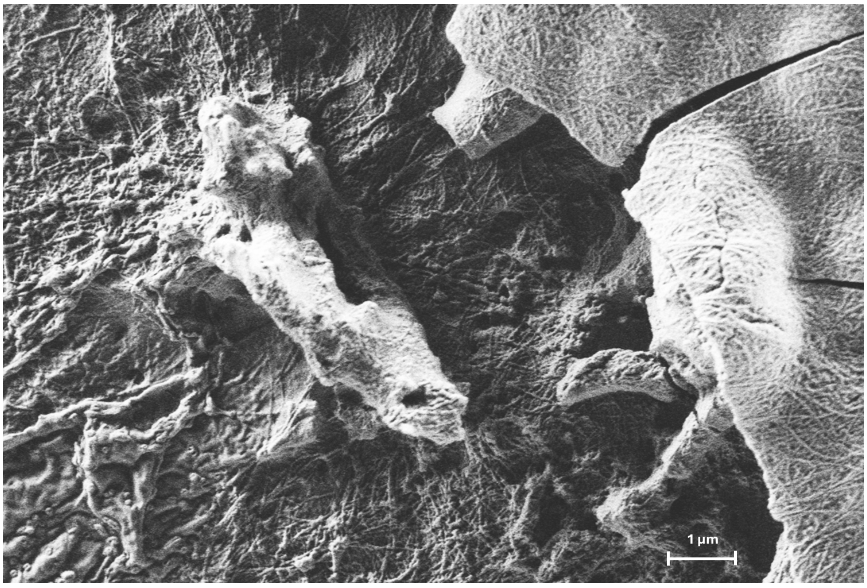

Figure S1.2 SEM image of insulin incubated for 140 minutes. On the right side of the image, the protein layer of aggregates with fibrils on the surface is particularly evident.

S2. FTIR data and PCA analysis of hyperbolic kinetics

Spectra of insulin undergoing hyperbolic aggregate formation are shown in Figure S2.1 panel a. Gaussian deconvolution of the amide I band is reported in panels b, c and d. The secondary structure percentages are reported in Table S2.1.

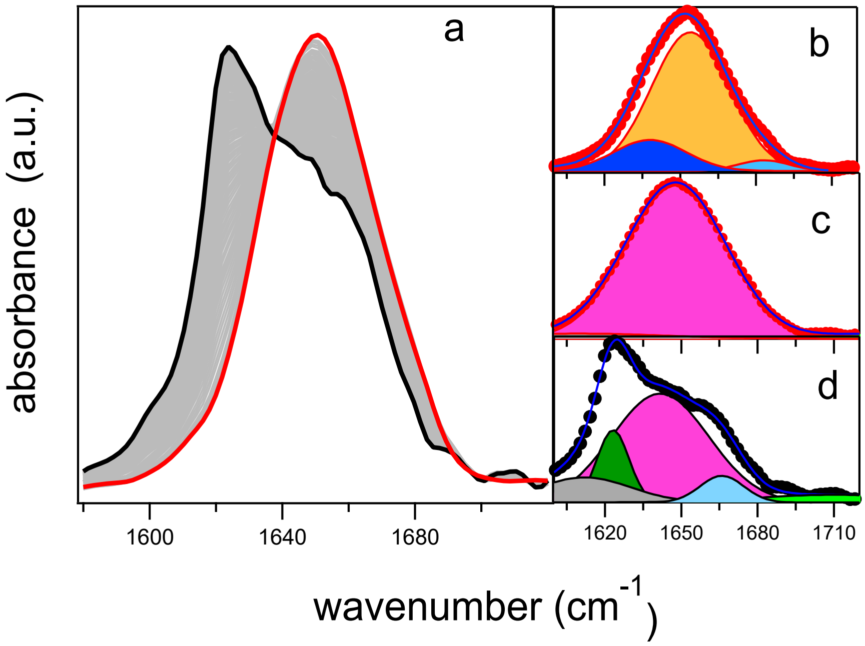

Figure S2.1 Spectra of insulin at different times of incubation (panel a). Red spectrum is collected at t= 0, the black one at the end on the incubation (t= 8h). In panels b, c and d the Gaussian deconvolution at t=0 (b), t=0.3 h (c) and t=8h (d) are reported.

Table S2.1. Secondary structure percentages (%) as obtained from the Gaussian deconvolution of the spectra in Figure S2.1. The assignment was performed according to the literature (see main text and references).

**assignment**	**t=0**	**t= 0.3 hours**	**t=8 hours**
Intermolecular β–sheet (1610-1615)	3	6	14
α-helix (1650-1655)	77		
β–sheet from dimers (1632-1639)	16		
β–sheet from fibrils (1622-1626)			14
Unordered structures from oligomers, Turns (1660-1685)	4		7
Random coil (1642-1649)		94	65

In Figure S2.2, the PCA outcomes for the second derivatives of data shown in Figure S2.1 are shown. The s_1 and s_2 scores (panel a) continuously vary to their asymptotic values. The corresponding loadings are reported in panels b and c of the same Figure.

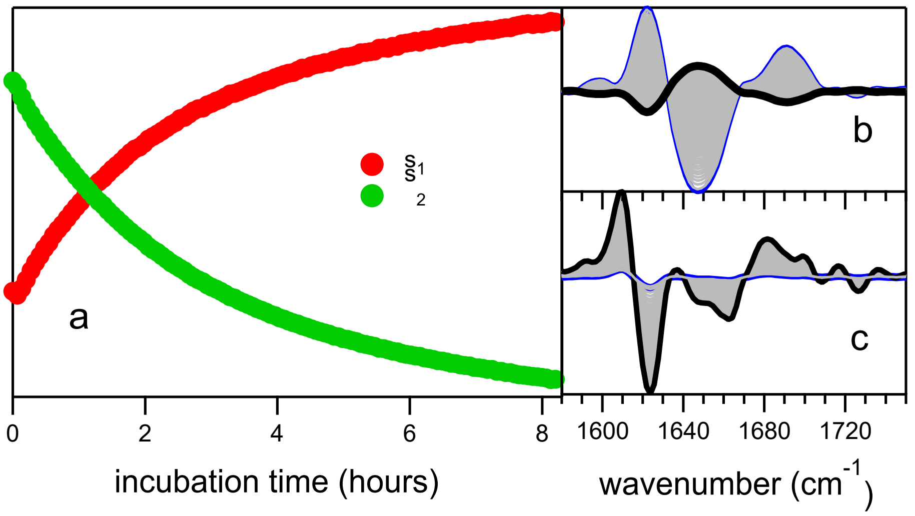

Figure S2.2. Panel a: scores s_1 and s_2. In panels b and c the corresponding loadings are also reported. Blue curves refer to the initial incubation time (t=0), black lines to the final time. Gray loadings correspond to the intermediate incubation times.

The $R(\omega, t)$ function is reported in the a and b panels of Figure S2.3. Panel a describes the abrupt initial transition from α-helical structure to random-coil. This process occurs concurrently with the loss of the β-structures at 1633 cm^{-1} and 1680 cm^{-1} due to dimer's disassembly. Panel b shows that at intermediate incubation times minor changes occur and mainly concern oligomeric structures (increase at 1666 cm^{-1}), turns and coils (decrease at 1675 cm^{-1}). For the longest times, t > 102 minutes, the $R(\omega, t)$ function does not provide further information, being null around zero.

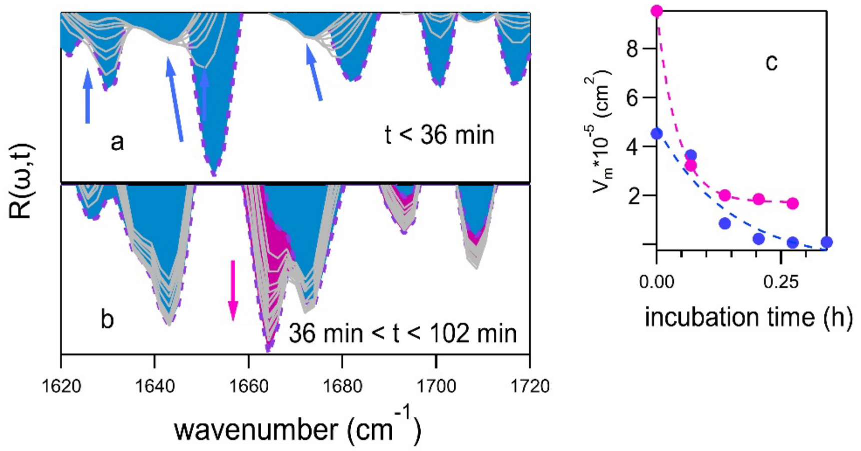

Figure S2.3. The $R(\omega, t)$ function in different time intervals (panels a and b). Arrows indicate the main changes of the secondary structure contributions: blue for those gradual decreasing, purple for the increasing ones. In panel c the time behavior in the first stage of incubation of the α-helix (purple) and the dimer contribution (blue) are reported.

S3. Estimate of the number of absorber from the band intensity

Assuming Gaussian shape for an absorption band, the integrated intensity I is related to the minimum of the second derivative V_m through the relation: $I = \sqrt{2\pi} V_m \sigma^3$, being σ the standard deviation.

According to the Lambert-Beer law, one can write:

$$\sqrt{2\pi} V_m \sigma^3 = C \cdot d \int_{\Delta\nu} \varepsilon(\nu)\, d\nu \quad \text{(s3.1)}$$

where C is the concentration of absorbers in Mole, d the optical path (cm) and ε(ν) the molar extinction coefficient of insulin ($cm^{-1}Mole^{-1}$). The integral is extended to a region $\Delta\nu = 3\sigma$ around the maximum. Within the approximation $\varepsilon(\nu = 1620\ cm^{-1})$= 9000 $M^{-1}cm^{-1}$ (reference [23] in the main text) the concentration C at any V_m writes:

$$C = \frac{\sqrt{2\pi} V_m \sigma^2}{3d\ \varepsilon(1620\ cm^{-1})} \qquad \text{(s3.2)}$$

The number of absorbers is then obtained for any V_m multiplying equation s3.2 for the volume of the solution (V_{IR}= 3.14*10^{-4} cm^3) and using d= 25*10^{-4} cm, σ= 5 cm^{-1}. Summing all values at different V_m, one has

$$N_{ABS} = \frac{C\ V_{IR}}{10^3} \cdot N_A = 3.2\ 10^{13}$$

where N_A is the Avogadro number.

In the same volume, the number of native monomers of insulin at 1mM/L is N= 4*10^{14}.

S4. Estimate of rate constant K_2

We assumed for the fibril and monomer concentrations the following expressions:

$$[m(x)] = m_0\left[1 - \frac{f(x)}{e^\alpha}\right] \qquad \text{(s4.1)}$$

$$[M(x)] = \frac{M_\infty}{e^\alpha} f(x) \qquad \text{(s4.2)}$$

where $x = t\,/\,T^*$, $f(x) = \frac{e^\alpha + 1}{1 + e^{\alpha(1-x)}} - 1$, α=T*/T_0, M_∞ the concentration of fibrils in the stationary phase and m_0 the initial monomer concentration.

The average of the V_m data in figure 7d of the main text has been expressed in Mole through equation s3. A fit to data with equation 4 of the text was implemented assuming *n*=2 and using an intensity factor A as free parameters. This latter is related to the rate constant K_2 through the expression:

$$K_2 = \frac{A e^\alpha K_D}{M_\infty m_0^2} \qquad \text{(s4.3)}$$

being K_D=(0.73 ± 0.03) μs^{-1} the rate of dissociation of the insulin dimers as obtained from an average of data in Ref. Acharya et al, J. Phys. Chem. B 125 9678−9691 (2021). The concentration M_∞ was inferred from the SEM data, assuming an average fibril volume V_f = (5.0 ± 1.5) 10^5 nm^3. Since the volume of the insulin monomer is estimated V_{ins}= 10.3 nm^3 and assuming only 10% of molecules forming fibrils, one obtains M_∞ = (2.5 ± 0.9) 10^{-9} Mole.
